# The use of cell salvage during second-stage reimplantation for the treatment of chronic hip periprosthetic joint infection: a retrospective cohort study

**DOI:** 10.1186/s13018-022-02955-3

**Published:** 2022-02-11

**Authors:** Zunhan Liu, Xuetao Yang, En-Ze Zhao, Xufeng Wan, Guorui Cao, Zongke Zhou

**Affiliations:** Department of Orthopedics, Orthopedic Research Institute, West China Hospital, West China Medical School, Sichuan University, #37 Guoxue Road, Chengdu, 610041 Sichuan Province People’s Republic of China

**Keywords:** Cell salvage, Periprosthetic joint infection, Second-stage reimplantation, Allogeneic blood transfusion, Reinfection

## Abstract

**Introduction:**

Given the possibility of inadvertent bacterial contamination of salvaged blood, the use of cell salvage is relatively contraindicated in cases of reimplantation for chronic hip periprosthetic joint infection (PJI). However, there are no published data supporting this assertion. The purpose of the current study was to compare the reinfection rate and rate of postoperative allogeneic blood transfusion (ABT) in second-stage reimplantation for PJI with or without intraoperative cell salvage reinfusion.

**Materials and methods:**

We identified 125 patients who underwent two-stage exchange for chronic hip PJI between November 2012 and April 2019. The groups of patients who had (*n* = 61) and had not (*n* = 64) received intraoperative cell salvage reinfusion were compared with respect to the curative infection-free rate. Moreover, we compared the need for postoperative ABT and identified independent factors associated with ABT using multiple regression analysis.

**Results:**

The log-rank survival curve with an endpoint of infection eradication failure was not significantly different between the cell salvage group (98.4%, 95% CI 95.3–99.9%) and the control group (95.3%, 95% CI 90.2–99.9%) at one year (log rank, *P* = .330). The rates of postoperative ABT in the cell salvage group were significantly lower than those in the control group (11.5% vs 26.6%, *P* = .041). In multivariable models, patient age, body mass index, preoperative hemoglobin level, and intraoperative cell salvage were independent predictors of ABT exposure (*P* < .05).

**Conclusions:**

The use of cell salvage during reimplantation in two-stage exchange for chronic hip PJI did not appear to increase the reinfection rate, while it significantly reduced the rate of postoperative allogeneic red blood transfusion. Greater age, lower BMI, lower preoperative hemoglobin, and non-intraoperative cell salvage reinfusion were associated with higher rate of allogeneic red blood transfusion.

## Introduction

Revision hip arthroplasty is a complex procedure that is associated with substantial perioperative blood loss and high allogeneic blood transfusion requirements [1]. However, allogeneic blood transfusion in revision total hip arthroplasty is associated with deleterious effects, including deep venous thrombosis, wound healing problems, and most importantly, an increased risk of periprosthetic joint infection (PJI) [2]. Numerous approaches are used to avoid allogeneic transfusions, including optimization of preoperative anemia, antifibrinolytic therapy, and the use of cell salvage systems [3–5]. Cell salvage offers an effective approach to reduce the need for allogeneic blood transfusion in primary total hip arthroplasty (THA) and aseptic revision [6]. However, the evidence for its use in patients undergoing two-stage PJI treatment remains unknown.

Historically, two-stage exchange arthroplasty using an antibiotic-loaded cement spacer is recognized as the “gold standard” for treating chronic PJI [7]. However, positive intraoperative cultures at second-stage reimplantation are reported to be found in up to 44% of patients [8], and the cumulative incidence of reinfection after two-stage exchange was 4% at 1 year and 14% at 5 years [9]. Given the possibility of persistent or undetectable infection and potential risk of bacterial dissemination, the use of cell salvage is relatively contraindicated in cases of hip revisions for PJI [10]. While several non-orthopedic studies specifically evaluated whether it is safe to reinfuse washed blood products in the setting of infection [11–13], their results demonstrated little, if any, evidence of bacterial dissemination from cell salvage devices [11, 12, 14, 15]. Otherwise, other orthopedic surgeons did not consider infection to be a contraindication to the use of cell salvage in the setting of revision hip surgery, which has sometimes been implemented in second-stage reimplantation for PJI [10, 16]. To date, no published data have been found in favor of or against the use of cell salvage in the setting of reimplantation for PJI. Compared with non-orthopedic surgeries, joint arthroplasty or revision is more demanding in terms of ensuring asepsis. Thus, the risks and benefits of using cell salvage during reimplantation must be weighed.

The objectives of the study were (1) to evaluate whether the use of cell salvage during reimplantation in two-stage exchange for PJI increases the reinfection rate, (2) to determine if the use of cell salvage during reimplantation decreases the need for postoperative allogeneic blood transfusion, and (3) to identify the potential factors associated with postoperative allogeneic blood transfusion. Our hypothesis was that the use of cell salvage during reimplantation for the treatment of chronic hip PJI would not increase the reinfection rate and may reduce the need for postoperative allogeneic blood transfusion.

## Materials and methods

### Study design and patients

Prior to conduction of the study, institutional Clinical Trials and Biomedical Ethics Committee approval was obtained. All patients knew the use of cell salvage was relatively contraindicated in cases of hip revisions for PJI and gave their written informed consent before reimplantation. We performed a single-center, retrospective, cohort study that enrolled consecutive patients who were treated for chronic hip PJI in a two-stage revision from November 1, 2012 to April 30, 2019, in which availability of second-stage culture results was mandatory. PJI was diagnosed according to the criteria published by the Musculoskeletal Infection Society and modified by the International Consensus Group as of 2014 [17], and chronic PJI was defined as any PJI present for more than 4 weeks from the index surgery [18]. Exclusion criteria comprised patients who (1) received intraoperative allogeneic blood transfusion or allograft bone transplantation during the second-stage reimplantation because these may increase the risk of reinfection and confound the analysis of reinfection sources; (2) had a known allergy to tranexamic acid (TXA), a history of arterial or venous thromboembolic event (e.g., stroke, pulmonary embolism, deep venous thromboembolism), coronary artery disease (placement of an arterial stent or myocardial infarction within the past six months), renal failure (serum creatinine > 200 mmol/l, creatinine clearance < 50 ml/min, dialysis), disseminated intravascular coagulation, hepatic failure, severe pulmonary disease; (3) had undergone preoperative anticoagulation therapy (excluding aspirin) or refusal of allogeneic blood transfusion; (4) treated with TXA in a way that deviated from the following standard practices in our institution from November 2012: TXA was routinely administrated a bolus (15 mg/kg) 5 min before incision, and administrated again if the duration of reimplantation exceeded 2 h. This study was conducted and reported in line with STROCSS 2019 criteria [19].

### Two-stage revision procedure

All revision procedures were performed using a classical posterolateral approach by 4 senior surgeons who specialized in total joint arthroplasty. During the first-stage revision procedure, thorough debridement was completed to excise sinus tracts, all infected tissues, and devitalized bone after removal of all foreign materials. Then, the wounds were repeatedly cleansed in 3% hydrogen peroxide for 4–5 min, irrigated with 0.9% saline, and finally cleansed in 2.5% povidone iodine for 4–5 min. This is the standard procedure in cases of PJI in our institution. Broad-spectrum antibiotics were administrated intravenously after the surgical fields were thoroughly irrigated with saline using a low-pressure system. Five to eight solid samples, both bone and soft-tissue, and a sample of synovial fluid were routinely obtained in the procedure and sent for microbiological culture and histological examination. All hips underwent implantation of an antibiotic-impregnated, handmade polymethylmethacrylate bone cement spacer containing 2 g of vancomycin and 1 g gentamicin per pack of Palacos cement (Biomet, Warsaw, IN).

Postoperative antibiotic administration protocol was under the guidance of infectious diseases specialists according to the antimicrobial susceptibility test. In all cases of PJI, medical therapy was initiated using broad-spectrum antibiotics (typically vancomycin and cefuroxime). If the tissue sample cultures were positive, organism-specific antibiotics were administered; otherwise, the empirical antibiotics strategy was vancomycin and cefuroxime. Patients received intravenous antibiotics for 6 weeks and oral antibiotics for 6 weeks following the first-stage revision. Second-stage reimplantation was considered when the general status of the patient was suitable and there were no signs of infection, which was confirmed by the downtrend of C-reactive protein and erythrocyte sedimentation rate to normal or near normal levels.

The second-stage reimplantation was performed under targeted prophylactic-guided antibiotics. Synovial fluid was routinely taken for bacterial culture before antibiotic administration. Frozen sections were also routinely performed to exclude residual infection. Frozen sections with greater than 10 white blood cells per high-power field were considered to have persistent infection; 5 to 9 white blood cells per high-power field were considered to have suspicious infection; and less than 5 white blood cells per high-power field were considered to eradicate infection [20]. After removal of the bone cement spacer, the joint cavity was cleansed in hydrogen peroxide and povidone iodine and irrigated with saline which was consistent with the procedure of the first-stage revision. All patients had cementless, porous-coated, diaphyseal engaging non-modular femoral stems.

According to the policy of our institution, we employed intraoperative cell salvage collection (3000P, Jingjing Medical Equipment, Beijing, China) for all second-stage revision hip procedures. Any fluid visibly contaminated by infection, metallic debris, and cement was not collected. All blood from operative fields and gauze was centrifuged with a leucocyte depletion filter (40 µm, Nanjing Shuangwei, Nanjing, China) and washed with 1000 mL of 0.9% sodium chloride, and then transferred to a sterile collecting bag. The volume of salvaged blood was standardized to hematocrit 55%. As per the result of hemoglobin and hematocrit measured intraoperatively using blood gas analysis, salvaged blood was processed to transfuse once the estimated blood loss exceeded 500 ml. The patients who did not have intraoperative cell salvage transfusion were included in the control group as their estimated blood loss was less than 500 ml.

Strict transfusion criterions were implemented in keeping with the clinical practice guidelines recommended from the American Association of Blood Bank [21], postoperative allogenic blood transfusion was only allowed for patients with a hemoglobin level < 70 g/l or with a hemoglobin level between 80 and 100 g/l in the setting of pre-existing cardiovascular disease, active bleeding, arterial thromboembolic event or sepsis, persistent symptoms despite adequate volume resuscitation. Plasma was transfused if more than four blood units were transfused or if coagulation parameters were out of acceptable ranges. When allogeneic blood transfusion was indicated, 1 unit of packed red blood cells was transfused to increase Hb levels to 8.0 g/dl.

Systemic intravenous antibiotics against the microorganism isolated at the first-stage revision were maintained until microbiological results were available. In general, if synovial cultures were positive, organism-specific antibiotics were administered for 4 weeks and then orally for 4 weeks. If no microorganisms were identified, intravenous antibiotic strategy was the same as that for the first-stage revision for one week and then orally for an additional 2 weeks. If a new microorganism was identified in more than 2 samples, organism-specific systemic antibiotics were administrated for 8 weeks.

### Assessments

We obtained demographic data as well as comorbidities, antibiotic administration, and culture results at the time point of reimplantation from all patients. McPherson’s host classification [22], patient-related risk factors (diabetes mellitus, inflammatory arthritis, chronic hepatitis, chronic anemia, tobacco use, alcoholism, resistant organism), component exchange, and positive culture are associated with an increase in reinfection rate. Hence, we obtained and compared these results between the 2 groups (Tables 1, 2).

**Table 1:** Comparison of demographic data between the 2 groups. Numbers indicate median (25th-75th interquartile range)

Variables	Cell salvage reinfused group	Control group		*P-*Value	
	(*n* = 61)	(*n* = 64)			
Gender, male *n* (%)	32 (52.5)	48 (75.0)		0.221^b^	
Age at reimplantation (year)	59.0 (49.5–70.0)	56.0 (45.3–69.0)		0.527^a^	
BMI (kg/m^2^)	24.7 (21.9–26.5)	24.1 (22.0–27.5)		0.947^a^	
ASA classification, *n* (%)				0.568^b^	
I–II	39 (63.9)	45 (70.3)			
III	22 (36.1)	19 (29.7)			
Charlson comorbidity index, *n* (%)				0.342^b^	
1–2	16 (26.2)	22 (34.4)			
3–5	27 (44.3)	30 (46.9)			
≥ 6	18 (29.5)	12 (18.8)			
Comorbidities, *n* (%)					
Diabetes mellitus	7 (11.5)	10 (15.6)		0.605^b^	
Inflammatory arthritis	4 (6.6)	6 (9.4)		0.744^b^	
Chronic hepatitis	3 (4.9)	5 (7.8)		0.718^b^	
Chronic anemia	8 (13.1)	11 (9.4)		0.622^b^	
Tobacco use, *n* (%)	19 (31.1)	28 (43.8)		0.196^b^	
Alcoholism, *n* (%)	21 (34.4)	25 (39.1)		0.711^b^	
Resistant strain, *n* (%)	6 (9.8)	6 (9.4)		1.000^b^	
Positive culture, *n* (%)	16 (26.2)	19 (29.7)		0.695^b^	
McPherson host type, *n* (%)				0.865^b^	
A	24 (39.3)	27 (42.2)			
B	25 (41.0)	27 (42.2)			
C	12 (19.7)	10 (15.6)			
Components revised, *n* (%)				0.380^b^	
Acetabular component	4 (6.6)	2 (3.1)			
Femoral component	0 (0.0)	2 (3.1)			
Dual components	57 (93.4)	60 (93.8)			
Time of antibiotic administration (week)					
Spacer stage duration (mo)	6.0 (4.5–11.0)	7.0 (5.0–11.8)		0.630^a^	
Follow-up (mo)	70.0 (54.5–94.0)	75.0 (46.3–84.8)		0.592^a^	
Failure after reimplantation, *n* (%)	1 (1.6)	3 (4.7)		0.330^c^	

*ASA* American Society of Anesthesiologists, *BMI* body mass index

^a^The Mann–Whitney *U* test

^b^The Fisher exact test

^c^The log-rank test

**Table 2:** Microbiological analysis of the synovial fluid culture during second-stage reimplantation

Pathogen, *n* (%)	Cell salvage reinfused group	Control group	Total
	(*n* = 61)	(*n* = 64)	(*n* = 125)
*Staphylococcus epidermidis*	5 (8.2)	7 (10.9)	12 (9.6)
*Staphylococcus aureus*	2 (3.3)	4 (6.3)	6 (4.8)
*Staphylococcus haemolyticus*	2 (3.3)	0 (0.0)	2 (1.6)
*Staphylococcus capitis*	1 (1.6)	2 (3.1)	3 (2.4)
*Staphylococcus warneri*	1 (1.6)	0 (0.0)	1 (0.8)
*Staphylococcus cohnii*	1 (1.6)	0 (0.0)	1 (0.8)
*Streptococcus agalactiae*	1 (1.6)	0 (0.0)	1 (0.8)
*Enterobacter cloacae*	1 (1.6)	1 (1.6)	2 (1.6)
*Pseudomonas aeruginosa*	1 (1.6)	2 (3.1)	3 (2.4)
*Bacillus cereus*	1 (1.6)	0 (0.0)	1 (0.8)
*Acinetobacter junii*	1 (1.6)	0 (0.0)	1 (0.8)
*Klebsiella oxytoca*	1 (1.6)	0 (0.0)	1 (0.8)
Polymicrobial (≥ 2 organisms)	1 (1.6)	1 (1.6)	2 (1.6)
Culture-negative	42 (68.9)	47 (73.4)	89 (71.2)

Clinical follow-up was conducted routinely at 3 weeks, 3 months, and 6 months after two-stage revision and annually thereafter until the final follow-up. The definition of a successful treated infection was based on the modified Delphi-based international multidisciplinary criteria [17]: (1) a healed wound, without a fistula, drainage, or pain and without recurrence caused by the same organism, (2) no subsequent surgical intervention for persistent or peri-operative infection, (3) no occurrence of PJI-related mortality; (4) no requirement for long-term (> 6 months) suppressive antibiotic treatment. If one of these criteria was not met, infection eradication failure was considered as established.

Furthermore, we analyzed whether the use of cell salvage during reimplantation led to a decreased rate or volume in postoperative allogeneic blood transfusion and identified the risk factors for allogeneic blood transfusion. We recorded preoperative and postoperative hemoglobin levels, preoperative and postoperative hematocrit levels, postoperative allogeneic blood transfusion (rates and units) within 7 days of reimplantation, and the amount of blood from cell salvage reinfused during reimplantation. The calculated blood loss = the patient’s blood volume (PBV) * (Hct_pre_—Hct_post_)/Hct_ave_ (Hct_pre_ refers to the preoperative hematocrit level. Hct_post_ refers to the minimum hematocrit level before transfusion. Hct_ave_ refers to the average of Hct_pre_ and Hct_post_) [23]. PBV = *k*1 * height (m^3^) + *k*2 * weight (kg) + *k*3 (*k*1 = 0.3669, *k*2 = 0.03219, and *k*3 = 0.6041 for men; and *k*1 = 0.3561, *k*2 = 0.03308, and *k*3 = 0.1833 for women) [19, 24]. All hemoglobin and hematocrit levels were measured routinely up to day 5 after the procedure.

### Statistical analysis

All statistical analyses were carried out using SPSS version 26 (IBM Corporation, Armonk, NY, USA). The normality of the distributions was tested for all scale variables by Kolmogorov–Smirnov’s test. Differences between groups were analyzed using Fisher’s exact test for categorical variables while continuous variables were compared by Mann–Whitney *U* test. Categorial variables are presented using counts and percentages, while continuous variables are summarized using the median with 25th and 75th percentiles. The primary analysis was to evaluate the influence of cell salvage during reimplantation by comparing the rate of infection eradication failure by Kaplan–Meier curve and log-rank test. Next, a multiple logistic regression was performed to identify independent variables associated with the need for allogeneic red blood transfusion. Covariates with a *P* value < 0.05 by univariate analysis were integrated stepwise into the multivariate regression model. For all comparisons, the level of statistical significance was set at *P* < 0.05. A post hoc power analysis (PASS, version 19.0) considered an alpha error probability of 0.05, a study power of 80%, a proportion of 30% of patients who did not receive intraoperative cell salvage reinfusion, and a relative risk of 2.5. The required sample size was 57 patients per group.

## Results

### Patients’ demographics

We initially identified a total of 141 patients with a minimum 2-year follow-up after reimplantation. Of these, patients who received intraoperative allogeneic blood transfusion (*n* = 4), those who received allograft bone transplantation (*n* = 3), those who had a history of deep venous thromboembolic event (*n* = 1), and those who did not follow the standard TXA practices for PJI in our institution (n = 7) were excluded. The final cohort consisted of 125 patients and was divided into 2 groups: patients who received intraoperative cell salvage reinfusion (*n* = 61) and those who did not receive intraoperative cell salvage reinfusion (*n* = 64), as the control group. Table 1 shows and compares baseline and perioperative characteristics between the cell salvage reinfused group and the control group; there was no significant difference observed between the 2 groups. The median follow-up time was 73.0 (53.0–89.0) months for all patients, 70.0 (54.5–94.0) months for the cell salvage group and 75.0 (46.3–84.8) months for the control group (*P* = 0.592). A total of 36 patients (28.8%) had ≥ 1 positive culture and 2 patients (1.6%) had ≥ 2 positive cultures at second-stage reimplantation (Table 2).

### Infection eradication rate

There were 4 patients who were considered to have infection eradication failure in this study, one in the cell salvage reinfused group and 3 in the control group. The reinfection of the 4 patients occurred within one year. The microbiological results of the synovial fluid culture of the 4 patients are summarized in Table 3. The one-year infection-free rate was 98.4% (95% confidence interval 95.3–99.9%) for the cell salvage reinfused group and 95.3% (95% confidence interval 90.2–99.9%) for the control group (log-rank, *P* = 0.330).

**Table 3:** Characteristics of the patients with infection eradication failure after two-stage reimplantation

No	Gender	Age	Cell salvage	Isolated microorganism in first-stage revision	Isolated microorganism in second-stage revision	Evidence of failure and microorganism
1	Male	56	No	Not found	*Staphylococcus epidermidis*	Repeated debridement
2	Female	63	Yes	*Staphylococcus aureus*	*Staphylococcus aureus*	Hip pain and requirement for another course of antibiotic treatment
3	Male	58	No	Not found	*Enterobacter cloacae*	Persistent infection and positive culture of *Enterobacter cloacae, Candida parapsilosis, and Citrobacter braakii*
4	Female	56	No	Not found	*Pseudomonas aeruginosa*	Repeated debridement and positive culture of *Pseudomonas aeruginosa*

### Postoperative allogeneic blood transfusion

In total, 24/125 (19.2%) patients received allogeneic blood transfusion up to 7 days after reimplantation: 7/61 (11.5%) in the cell salvage reinfused group, and 17/64 (26.6%) in the control group. In relation to the proportion, the postoperative allogeneic red blood transfusion rate was significantly lower in patients who received cell salvage reinfusion than in the control group (11.5% vs 26.6%, *P* = 0.041). However, there was no significant difference observed in the rate of postoperative fresh frozen plasma transfusion between the 2 groups (6.6% vs 7.8%; *P* = 1.000). When comparing the volume of postoperative allogeneic red blood and fresh frozen plasma per case, there was also no significant difference between the 2 groups (2.0 units vs 1.5 units, *P* = 0.630; 300 ml vs 200 ml, *P* = 0.896). The majority of allogeneic red blood (14/25, 56.0%) was transfused on day 1. The calculated blood loss was significantly lower in patients in whom cell salvage was set up than in controls (846 ml vs 1195 ml, *P* = 0.002). The clinical results related to postoperative allogeneic blood transfusion are shown in Table 4.

**Table 4:** Clinical data for second-stage reimplantation. Numbers indicate median (25th–75th percentile)

Viable	Cell salvage reinfused group	Control group	*P-*value
	(*n* = 61)	(*n* = 64)	
Hemoglobin (g/l)			
Preoperative	137 (130–142)	136 (131–143)	.694^a^
Day 1	108 (99–117)	105 (94–112)	.112^a^
Hematocrit (%)			
Preoperative	41 (40–42)	41 (39–43)	.919^a^
Day 1	32 (27–35)	32 (28–35)	.804^a^
Estimated blood loss (ml)	846 (623–1230)	1195 (861 to 1474)	.002^a^
Reinfused blood (ml)	300 (200–750)	–	
Allogeneic RBC transfused (units)	2.0 (1.5–2.0)	1.5 (1.5–2.0)	.630^a^
Allogeneic RBC transfused, *n* (%)	7 (11.5%)	17 (26.6%)	.041^b^
Plasma transfused (ml)	300 (163–1712)	200 (200–400)	.896^a^
Plasma transfused, *n* (%)	4 (6.6%)	5 (7.8%)	1.000^b^
Duration of operation (min)	159 (143–180)	156 (130–175)	.128^a^
Length of hospitalization (days)	11 (9–14)	12 (10–18)	.045^a^

^a^The Mann–Whitney *U* test

^b^The Fisher exact test

### Factors associated with postoperative allogeneic red blood transfusion

Univariate logistic regression analysis showed that age, BMI, preoperative hemoglobin, and intraoperative cell salvage reinfusion were associated with the rate of postoperative allogeneic red blood transfusion (*P* = 0.031, 0.004, 0.005, and 0.037, respectively). After adjustment for covariates with the use of the multivariate logistic model, greater age (hazard ratio (HR) 3.092, 95% CI 1.048–9.123, *P* = 0.041), lower BMI (HR 0.805, 95% CI 0.687–0.944, *P* = 0.007), lower preoperative Hb (HR 0.092, 95% CI 0.018–0.477, *P* = 0.004), and non-intraoperative cell salvage reinfusion (HR 0.176, 95% CI 0.051–0.608, *P* = 0.006) were identified as independent factors associated with a higher rate of postoperative allogeneic red blood transfusion. The results of the binary logistic regression analysis are summarized in Table 5.

**Table 5:** Factors associated with postoperative allogeneic blood transfusion rate

Coefficient	Univariate Logistic regression			Multivariate logistic regression		
	HR	*P* value	95% CI	Adjusted HR	*P* value	95% CI
Age (year)						
< 60	Reference	Reference	Reference	Reference	Reference	Reference
≥ 60	2.810	.031	1.101–7.166	3.092	.041	1.048–9.123
BMI (kg/m^2^)	0.808	.004	0.698–0.935	0.805	.007	0.687–0.944
Preoperative hemoglobin (g/l)						
< 120	Reference	Reference	Reference	Reference	Reference	Reference
≥ 120	0.156	.005	0.043–0.567	0.092	.004	0.018–0.477
Preoperative hematocrit (%)						
< 45	Reference	Reference	Reference	NA	NA	NA
≥ 45	0.218	.270	0.034–2.163	NA	NA	NA
Intraoperative cell salvage						
No	Reference	Reference	Reference	Reference	Reference	Reference
Yes	0.358	.037	0.137–0.939	0.176	.006	0.051–0.608

*HR* hazard ratio, *NA* not applicable

*P* < .05, statistically significant difference

## Discussion

In our series of chronic hip PJIs managed with a two-stage revision protocol, we found an infection eradication failure rate of 3.2%. Receiving cell salvage during reimplantation did not increase the reinfection rate in our study. The use of cell salvage during reimplantation reduced the need for postoperative allogeneic red blood transfusion, confirming our hypothesis. Greater age, lower BMI, lower preoperative hemoglobin, and non-intraoperative cell salvage reinfusion were identified as independent factors associated with higher rate of postoperative allogeneic red blood transfusion (Table 5).

Reinfection is one of the most common complications after two-stage revision of PJI with severe medical and economic impacts on the affected patients and healthcare budgets [25]. Prior studies have been published regarding risk factors for reinfection after two-stage revision [26, 27], but no data have addressed cell salvage during reimplantation. Considering that contaminated blood caused by persistent or undetected infection may lead to bacteremia, sepsis, or the possibility of reinfection after revision, the use of cell salvage is relatively contaminated in cases of septic revisions [10]. However, several non-orthopedic studies have specifically evaluated whether it is safe to reinfuse these products in infection circumstances; Ozmen et al. [28] found that patients receiving washed blood collected from the peritoneal cavity during abdominal trauma exploration did not have increased wound infection compared with those who received bank blood, and Bowley et al. [29] reported that cell salvage during laparotomy for penetrating abdominal trauma did not increase the postoperative reinfection rate. Although several orthopedic surgeons also did not consider PJI as a relative contraindication to cell salvage which has sometimes been implemented in second-stage reimplantation, no evidence has been found in favor of or against the use of cell salvage during reimplantation. In the present study, we found similar results to previous non-orthopedic studies in which the use of cell salvage during reimplantation did not appear to increase the reinfection rate. One possible reason could be that almost all persistent bacteria during reimplantation were removed by a combination of cell washing and leukocyte depletion filtering. Waters et al. [14] showed that the combination of cell salvage washing and leukocyte depletion filtration resulted in a 97.6 to 100% removal of bacterial loads. Another possible reason may be that the reinfection rate after second-stage reimplantation in our cohorts was significantly lower than that in previous studies [9, 26, 30, 31], which might be insufficient to detect significant differences.

Few absolute contraindications have been clearly proposed for cell salvage [10]. Due to end-organ damage as a result of administering lysed red blood cells, anything that results in red cell lysis or destruction is defined as a definite or absolute contraindication [32]. In terms of blood contamination caused by undetected infection of PJI, reinfusion of such blood is of theoretical risk of reinfection. In fact, bacterial contamination of salvaged blood appears to be common [11, 33]. Previous studies have demonstrated that cell salvage can be used safely in the face of bacterial contamination and noted that washing and filtration can remove bacterial contaminants up to a concentration of 10^3^ cfu/ml [14]. For patients undergoing second-stage reimplantation, bacterial loads of positive cultures in the blood are low concentration after administering systematic antibiotics and strictly following the indications for second-stage reimplantation. Thus, we consider that the use of cell salvage during second-stage reimplantation for PJI will not increase the reinfection rate.

There is ongoing discussion about the effect of cell salvage on the need for additional allogeneic blood transfusion [34]. Previous studies demonstrated that the use of cell salvage significantly decreased the allogeneic transfusion requirement in aseptic hip revision [34, 35]. However, unlike aseptic hip revision, procedures performed for PJI are associated with more complex surgical techniques and higher blood loss [16]. In our cohorts, we found that patients receiving cell salvage during reimplantation had a significantly lower exposure rate of postoperative allogeneic red blood transfusion, but a similar volume of allogeneic red blood transfusion. These findings are in line with the results of aseptic hip revisions reported in former studies [34, 35]. However, the rate of postoperative allogeneic transfusion in the cell salvage group (11.5%) was significantly lower than the previously reported rates, of 37% and 55% [34, 35]. A lower allogeneic transfusion rate may demonstrate development in patient blood management, including the use of antifibrinolytic agents (such as TXA), more efficient cell salvage, and improvement in the surgical technique of revision for PJI.

We found that patients with greater age, lower BMI, lower preoperative Hb (< 120 g/l), and non-receiving transfusion were more likely to receive postoperative allogeneic blood transfusion. These findings were similar to the results of primary THA and aseptic hip revisions [36–38]. In a large cohort study by Adam et al. [36], age (HR per ten years = 10.1) and BMI of ≤ 30 kg/m^2^ (HR = 1.4) were found to be significant predictors for receiving allogeneic transfusion after THA. Slover et al. [37] reported, in a statewide database, similar results that greater age had a significantly higher odds of red blood transfusion after THA. Walsh et al. reported that preoperative hemoglobin level (HR = 0.35) was an independent predictor of postoperative allogeneic blood transfusion after aseptic hip revision [38]. Importantly, the use of cell salvage had a protective effect on the risk of postoperative allogeneic blood transfusion, suggesting that the use of cell salvage during reimplantation for PJI was clinically beneficial. Our study justified the routine use of cell salvage during reimplantation for PJI patients with greater age and lower BMI, and strongly supported the use for patients with preoperative hemoglobin less than 120 g/l.

While this is the first study evaluating the safety and efficacy of cell salvage during second-stage reimplantation by a standardized diagnostic and therapeutic algorithm for PJI, it has several limitations. First, the retrospective design decreased the level of evidence and implies selection and recall bias. Second, the small sample size, especially the relative infrequency of reinfection rate (3.2%), might be underpowered to reveal differences in the use of cell salvage and predict risk factors for reinfection after surgery. Therefore, the effect of the use of cell salvage during reimplantation on the reinfection rate in this study needs to be interpreted with caution and studies with larger sample sizes are needed. Third, four different surgeons used cell salvage during second-stage reimplantation, which inevitably led to variability. However, each of the 4 surgeons had a high volume of PJI cases every year, performed similar surgical techniques, and followed the same antibiotic protocols in treating patients with PJIs.

## Conclusion

The use of cell salvage during reimplantation in two-stage exchange for chronic hip PJI did not appear to increase the reinfection rate, while it significantly reduced the rate of postoperative allogeneic red blood transfusion. Greater age, lower BMI, lower preoperative hemoglobin, and non-intraoperative cell salvage use were identified as risk factors for higher rate of postoperative allogeneic red blood transfusion.

## Data Availability

Please contact author for data requests.

## Abbreviations

PJI: Periprosthetic joint infection
ABT: Allogeneic blood transfusions
THA: Total hip arthroplasty
TXA: Tranexamic acid
PBV: Patient’s blood volume
PASS: Post hoc power analysis
BMI: Body mass index
HR: Hazard ratio
